# Advances in understanding intestinal microbiota mechanisms and intervention strategies for anxiety, depression, sleep disorders, and constipation

**DOI:** 10.3389/fmicb.2026.1838417

**Published:** 2026-06-25

**Authors:** Xiaomeng Li, Wei Wang, Yu Guo, Chu Feng, Yinlu Fan, Yao Lu, Yan Lv, Yanhong Wang, Xunlei Pang

**Affiliations:** 1 Department of Gastroenterology, Affiliated Hospital of Xuzhou Medical University, Xuzhou, Jiangsu, China; 2 Department of Gastroenterology, The First People’s Hospital of Sihong County, Suqian, Jiangsu, China; 3 Department of Clinical Laboratory, Affiliated Hospital of Xuzhou Medical University, Xuzhou, Jiangsu, China

**Keywords:** anxiety, chronic constipation, depression, gut microbiota, microbiota–gut–brain axis, sleep disorders

## Abstract

Anxiety, depressive symptoms, sleep disorders, and chronic constipation frequently co-occur and collectively impose a substantial clinical burden. Although these conditions may involve partially overlapping neural, endocrine, immune, and metabolic processes, their shared pathophysiological basis has not been fully established. This narrative review examines the potential role of the microbiota–gut–brain axis in linking affective symptoms, sleep disturbances, and chronic constipation. Evidence from human observational studies, Mendelian randomization analyses, animal models, and preliminary interventional studies suggests that alterations in gut microbial composition and function may contribute to these clinical associations through microbial metabolites, immune signaling, neuroendocrine regulation, and neural pathways. The review also summarizes microbiota-targeted interventions, including probiotics, prebiotics, dietary modification, and fecal microbiota transplantation. However, substantial heterogeneity in study populations, microbial findings, experimental methods, and intervention protocols limits causal interpretation and clinical generalization. A more rigorous distinction among associative, mechanistic, genetic, and interventional evidence is therefore required when evaluating the therapeutic potential of microbiota-based strategies.

## 1 Introduction

Anxiety, depression, sleep disorders, and chronic constipation are common clinical conditions that frequently coexist within the same individuals or patient populations. Their co-occurrence suggests that these conditions may share partially overlapping pathophysiological processes and interact through bidirectional pathways, although no single established mechanism can explain all affected individuals (Wang J.-X. et al., 2025). Growing interest in communication between the intestinal environment and the central nervous system has provided a broader framework for examining these clinical associations. Within the gut–brain axis, neural, immune, endocrine, and metabolic signals transmit information bidirectionally, while central nervous system activity can influence intestinal motility, secretion, barrier function, and microbial homeostasis (Chandramowlishwaran et al., 2020). From a neurobiological perspective, anxiety and depression involve alterations in stress-responsive neural circuits, neurotransmitter systems, neuroendocrine regulation, neuroinflammation, and synaptic plasticity. Chronic stress has been associated with changes in glutamatergic neurotransmission and AMPA receptor-related signaling within the prefrontal cortex, hippocampus, and amygdala, which may contribute to maladaptive emotional and behavioral responses (Lee et al., 2022). Sleep disorders similarly involve disturbances in circadian regulation, sleep homeostasis, arousal systems, and stress-responsive pathways. Stress and insomnia can reinforce one another through glucocorticoid signaling and interactions among neuronal, glial, and neuroendocrine processes (Birch and Vanderheyden, 2022).

Chronic constipation is also influenced by coordinated neural and gastrointestinal mechanisms. The enteric nervous system, enterochromaffin cell-derived 5-hydroxytryptamine (5-HT), interstitial cells of Cajal, smooth muscle activity, and autonomic regulation jointly contribute to colonic transit and secretion. Psychological stress and altered central autonomic output may further affect visceral sensitivity, intestinal motility, and defecatory function. These mechanisms support the possibility that partially overlapping neurobiological, gastrointestinal, and microbiota-related pathways contribute to the observed clinical co-occurrence of affective symptoms, sleep disturbances, and constipation.

Against this neurobiological background, increasing attention has been directed toward microbial metabolites and their involvement in bidirectional gut–brain communication. Short-chain fatty acids (SCFAs), tryptophan-derived metabolites, bile acids, and other neuroactive compounds may influence neurotransmitter-related, immune, metabolic, and neuroendocrine processes, thereby linking intestinal activity with emotional regulation and circadian physiology (Lu et al., 2022; Quillin et al., 2021). Clinical studies in anxiety and depression have reported reduced microbial α-diversity, lower abundance of selected butyrate-producing taxa, and enrichment of taxa associated with pro-inflammatory profiles. However, these findings vary across populations and are influenced by diet, medication exposure, geographic background, diagnostic criteria, sample type, and sequencing methods (Liu et al., 2024). Microbial and metabolic alterations have also been associated with melatonin-related and circadian processes in sleep disorders (Tian et al., 2022). In chronic constipation, reported changes in taxa such as Bacteroides, Roseburia, and Clostridium have been linked to altered microbial metabolism, enteric nervous system activity, and gastrointestinal motility. These findings do not define a universal disease-specific microbial signature, but they support recurring functional themes involving SCFA production, barrier integrity, inflammatory signaling, tryptophan metabolism, and bile acid signaling. Microbiota-targeted strategies, including probiotics, prebiotics, dietary modification, and fecal microbiota transplantation (FMT), have therefore been investigated for emotional, sleep-related, and gastrointestinal symptoms. Their effects remain strain-, population-, disease-, and protocol-dependent, but selected studies indicate that these approaches may provide adjunctive benefits in appropriately defined clinical settings (Zhang J. et al., 2025; St-Onge and Zuraikat, 2019).

Clinical trials of microbiome-modulating interventions for anxiety disorders have produced heterogeneous results. Improvements have been reported in selected longitudinal and interventional studies, whereas several controlled trials have not demonstrated a clear advantage over placebo. Microbiota-based interventions should therefore be regarded as exploratory or adjunctive rather than established stand-alone treatments for anxiety disorders (Adıgüzel et al., 2025). Future studies should define disease-specific mechanisms, effective strains or intervention components, dose, treatment duration, baseline microbial characteristics, and patient subgroups that are most likely to respond.

Accordingly, this narrative review summarizes the biological communication pathways of the microbiota–gut–brain axis and evaluates microbiota-related evidence in anxiety and depression, sleep disorders, and chronic constipation. It further examines the clinical co-occurrence of these conditions and reviews microbiota-targeted interventions, including probiotics, prebiotics, dietary strategies, and FMT. Particular attention is given to the distinction among animal models, human observational studies, Mendelian randomization analyses, and interventional clinical studies because these designs provide different levels of evidence for biological plausibility, clinical association, causal inference, and therapeutic efficacy.

## 2 Literature search strategy

This article was developed as a narrative review. Relevant publications were identified through targeted electronic literature searches, primarily using PubMed, with supplementary retrieval of full-text articles and bibliographic information from journal and publisher websites. The final reference set mainly covered studies published between 2011 and 2025. Search concepts included combinations of terms related to “gut microbiota,” “gut microbiome,” “microbiota–gut–brain axis,” “anxiety,” “depression,” “sleep disorders,” “insomnia,” “chronic constipation,” “short-chain fatty acids,” “tryptophan metabolism,” “probiotics,” “prebiotics,” “dietary intervention,” and “fecal microbiota transplantation.”

Publications were considered when they addressed at least one of the following areas: (1) neural, immune, endocrine, metabolic, or gastrointestinal pathways involved in microbiota–gut–brain communication; (2) gut microbial composition or microbial metabolic alterations associated with anxiety and depression, sleep disorders, or chronic constipation; (3) clinical co-occurrence or potential bidirectional relationships among these symptom domains; or (4) the mechanisms, efficacy, safety, or limitations of microbiota-targeted interventions. The evidence base included systematic reviews and meta-analyses, human observational studies, Mendelian randomization analyses, animal and other preclinical studies, and interventional clinical studies. Publications that were outside the predefined scope, duplicated the same evidence without adding relevant information, or did not provide sufficient methodological detail for interpretation were not included in the narrative synthesis.

Peer-reviewed publications were prioritized. When a preprint was retained because it addressed an emerging mechanistic question for which no peer-reviewed alternative was available, it was explicitly identified as preliminary evidence and was not assigned the same evidential weight as peer-reviewed human or interventional research. Literature collection and initial analysis were performed by X. L., with W. W. contributing to literature screening. Because this article was designed as a broad narrative synthesis across heterogeneous clinical and experimental fields, no formal systematic review protocol, PRISMA flow diagram, quantitative meta-analysis, or structured risk-of-bias assessment was undertaken.

## 3 Fundamental concepts of the gut microbiota and the microbiota–gut–brain axis

### 3.1 Composition and functions of the gut microbiota

The human gut microbiota is a complex ecosystem composed of bacteria, fungi, viruses, archaea, and other microorganisms, with the colon containing the highest microbial density. In healthy adults, Firmicutes and Bacteroidetes are generally dominant bacterial phyla, followed by Actinobacteria and Proteobacteria, although their relative abundance varies substantially among individuals (Hrncir et al., 2021). Gut microorganisms ferment indigestible dietary substrates to generate SCFAs, including acetate, propionate, and butyrate, which contribute to host energy metabolism, immune regulation, and intestinal barrier maintenance (Lu et al., 2022). The gut microbiota also participates in the biosynthesis of vitamin K and several B vitamins and contributes to colonization resistance through competition for nutrients and modulation of the local intestinal environment (Tarracchini et al., 2024). In addition, microbial metabolism can influence the availability of neuroactive compounds and their precursors, including GABA- and 5-HT-related metabolites, thereby providing potential routes of communication with the nervous system (Quillin et al., 2021).

### 3.2 Bidirectional communication mechanisms of the gut–brain axis

The gut–brain axis is a complex bidirectional communication network involving neural, endocrine, immune, and metabolic pathways. The vagus nerve is a major neural route through which mechanical and chemical signals from the gastrointestinal tract are transmitted to the nucleus tractus solitarius (NTS) and subsequently integrated within higher brain regions, including the hypothalamus and amygdala (Longo et al., 2023). Endocrine signaling complements this neural pathway through the release of peptide hormones such as glucagon-like peptide-1 (GLP-1) and peptide YY (PYY) from enteroendocrine L cells. These hormones can act through peripheral neural pathways, circulating receptors, and brain regions with relatively permeable vascular interfaces to influence appetite, energy balance, and related behavioral processes (Gwak and Chang, 2021; Kim et al., 2018).

Immune signaling represents another important component of the microbiota–gut–brain axis. Intestinal barrier disruption and microbial imbalance may promote activation of immune cells in the lamina propria and increase the release of pro-inflammatory mediators, including interleukin-6 (IL-6) and tumor necrosis factor-α (TNF-α). These mediators may influence central processes through systemic circulation, endothelial and barrier-related mechanisms, or vagal afferent signaling (Gwak and Chang, 2021). Microbial metabolites may also regulate host gene expression through receptor-dependent and epigenetic pathways. Secondary bile acids and indole derivatives, for example, can interact with the aryl hydrocarbon receptor (AhR), whereas SCFAs may affect histone deacetylase (HDAC) activity. These pathways provide plausible mechanisms through which microbial metabolism may influence neuronal, glial, and immune function, although their relative importance differs across experimental and clinical contexts (Srivastava and Gupta, 2025; Qian et al., 2024).

### 3.3 Major pathways through which the gut microbiota may influence the nervous system

**Figure 1:**
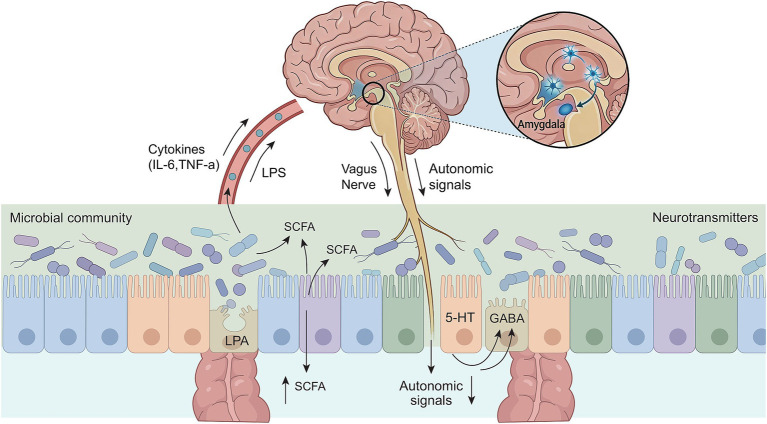
Signaling pathways of the microbiota–gut–brain axis. The figure illustrates neural, immune, metabolic, and neurotransmitter-related communication among the gut microbiota, intestinal epithelium, vagus nerve, and central nervous system. IL-6, interleukin-6; TNF-α, tumor necrosis factor-α; LPS, lipopolysaccharide; SCFA, short-chain fatty acid; LPA, lysophosphatidic acid; 5-HT, 5-hydroxytryptamine; GABA, γ-aminobutyric acid. The figure was independently drawn and integrated by the authors using Adobe Illustrator (Adobe Inc., San Jose, CA, United States), following commonly used schematic representations of medical and biological organs, cells, and signaling pathways. The figure provides a conceptual synthesis of the evidence reported in the cited literature. Arrows indicate reported or proposed biological relationships and do not imply that all depicted relationships represent established direct causality in human studies.

The gut microbiota may influence nervous system function through several interacting pathways. (1) Metabolic signaling: microbiota-derived SCFAs, including butyrate, can affect intestinal barrier integrity, immune responses, and epigenetic regulation. Experimental studies have also linked SCFA signaling with BDNF expression and hippocampal neuroplasticity (Li C. et al., 2025; Rathour et al., 2023); (2) Neurotransmitter-related pathways: selected microbial taxa can produce or modify GABA, catecholamine-related compounds, and tryptophan metabolites. Rather than directly supplying central neurotransmitters, these microbial activities may alter precursor availability, enteroendocrine signaling, vagal activity, and the kynurenine pathway (Rathour et al., 2023); (3) Microglial development and immune surveillance: germ-free animal models exhibit altered microglial morphology and immune responsiveness, while SCFA administration can partially restore selected homeostatic features. These findings provide mechanistic evidence in animal models but should not be directly equated with human psychiatric disorders (Blair et al., 2025). (4) HPA axis regulation: antibiotic exposure and microbial manipulation in animal models can alter anxiety-like behavior, cognition, hippocampal BDNF expression, and hypothalamic neuropeptide signaling (Foster et al., 2017). In human inflammatory depression, reduced abundance of selected SCFA-producing taxa and enrichment of inflammatory microbial profiles have been associated with TLR4/NF-κB- and NLRP3 inflammasome-related signaling. These findings support an interaction among microbial function, immune activation, neuroendocrine regulation, monoaminergic signaling, and oxidative stress, while the direction and magnitude of these relationships require disease- and population-specific interpretation (Liu et al., 2024) (Figure 1).

## 4 Gut microbiota-related evidence across specific disorders and mechanisms

### 4.1 Gut microbiota in anxiety and depression

#### 4.1.1 Characteristics of the gut microbiota in patients with anxiety and depression

A growing body of literature has described differences in gut microbial composition between individuals with anxiety or depression and comparison groups. Systematic reviews have reported recurrent enrichment of selected inflammation-associated taxa, including Enterobacteriaceae and Desulfovibrio, together with lower abundance of selected butyrate-producing taxa such as Faecalibacterium (Simpson et al., 2021). Studies in Chinese populations have additionally reported higher abundance of Actinobacteria, Proteobacteria, Rikenellaceae, and Porphyromonadaceae and lower abundance of Firmicutes, Prevotellaceae, and Ruminococcaceae in depressive phenotypes. Anxiety-related studies have reported lower levels of Lachnospira and Faecalibacterium and higher levels of Bacteroidetes, Enterobacteriaceae, and Fusobacterium in selected cohorts (Cao et al., 2025).

These taxonomic findings should be interpreted cautiously because α- and β-diversity results and the direction of individual bacterial changes are not consistent across studies. Heterogeneity may arise from differences in diagnostic criteria, symptom severity, psychotropic medication, diet, probiotic exposure, age, sex, body mass index, geographic background, sample size, stool versus mucosal sampling, 16S rRNA variable regions, shotgun metagenomic sequencing, reference databases, and bioinformatic pipelines. Cross-sectional studies also cannot determine whether microbial alterations precede affective symptoms or occur as a consequence of disease-related changes in diet, behavior, medication, and physiology. Although no single taxonomic signature has been consistently reproduced across populations, recurrent functional patterns include altered SCFA-producing capacity, inflammatory signaling, intestinal barrier-related changes, and tryptophan metabolism (Simpson et al., 2021).

#### 4.1.2 Correlation between gut microbiota metabolites and neurotransmitters

Gut microbial metabolism may influence neurotransmitter-related processes through SCFAs, tryptophan metabolites, bile acids, and enteroendocrine signaling. Butyrate has been associated with intestinal and blood–brain barrier integrity, immune regulation, HDAC inhibition, and BDNF-related neuroplasticity in experimental studies (Li C. et al., 2025). In human studies, an elevated kynurenine-to-tryptophan ratio has been associated with greater depressive symptom severity and may reflect reduced availability of tryptophan for 5-HT synthesis together with increased immune activation (Fatimi et al., 2025). Selected Bifidobacterium and Lactobacillus strains may also influence enterochromaffin signaling, GABA receptor expression, vagal pathways, and microbial metabolite production; however, these effects are strain- and model-specific (Bravo et al., 2011; Huang and Wu, 2021). Animal studies provide additional mechanistic evidence. Maternal or donor microbiota associated with depressive phenotypes can transfer selected anxiety- and depression-like behavioral features to recipient animals. Such FMT experiments support a contributory role for microbiota-associated factors in animal behavioral phenotypes, but they do not independently identify the responsible metabolites or establish direct causality in human psychiatric disorders (Zhang et al., 2024). Secondary bile acid signaling may also participate in emotional regulation. For example, TGR5-related pathways have been associated with microbiota-dependent anxiety- and depression-like behaviors in experimental models (Tao et al., 2024).

#### 4.1.3 Mechanisms of the microbiota–gut–brain axis in emotional regulation

The microbiota–gut–brain axis supports bidirectional communication through neural, immune, endocrine, and metabolic pathways. The vagus nerve can transmit intestinal sensory and microbial metabolite-related signals to the nucleus tractus solitarius and connected limbic and hypothalamic regions (Vasilyeva, 2023). Human observational studies have reported higher circulating LPS-related markers and pro-inflammatory cytokines, including IL-6 and TNF-α, in selected patients with anxiety or depression. These changes may be associated with barrier dysfunction, endothelial signaling, microglial activation, and reduced hippocampal neuroplasticity, although the sequence of these events has not been established in humans (Kumar et al., 2023).

HPA axis dysregulation represents another relevant pathway. Selected probiotic interventions have been associated with changes in cortisol or stress-related outcomes, but the effects vary according to strain, population, and study design (Fatimi et al., 2025). Maternal depression-related microbial changes have also been linked in animal studies with epigenetic and BDNF-related alterations in offspring (Zhang et al., 2024). Mendelian randomization analyses provide a complementary form of evidence by evaluating genetically predicted microbial traits. Selected analyses have identified potential protective associations for Bifidobacteriales and Bifidobacteriaceae and risk-related associations for *δ*-Proteobacteria and Desulfovibrionales. These findings provide genetic evidence consistent with potential causal relationships, but they remain dependent on instrument validity, taxonomic resolution, and the assumptions of Mendelian randomization (Lai and Xiong, 2025).

### 4.2 Gut microbiota and sleep disorders

#### 4.2.1 Alterations in intestinal microbiota among patients with sleep disorders

Studies of insomnia, sleep apnea, and experimental sleep deprivation have reported alterations in microbial diversity, taxonomic composition, and metabolic function. Human observational studies have described lower abundance of selected SCFA-producing taxa, including Faecalibacterium and Roseburia, together with enrichment of taxa associated with inflammatory profiles in some cohorts (Han et al., 2022). In mice, chronic sleep deprivation has been associated with reduced Lachnospiraceae, increased Tannerellaceae, and changes in microbial metabolic potential (Zhang et al., 2023). However, the direction and reproducibility of individual taxa differ across studies because of variation in sleep-disorder definitions, diet, medication, circadian timing of sample collection, host characteristics, and sequencing methods. These findings therefore support an association between sleep disruption and altered microbial ecology rather than a universal microbiota signature for sleep disorders.

#### 4.2.2 Influence of intestinal microbiota on sleep–wake rhythms

The gut microbiota may participate in circadian and sleep–wake regulation through microbial metabolites, neuroendocrine signaling, immune activity, and feeding-related rhythms. SCFAs and tryptophan-derived metabolites can influence enteroendocrine and 5-HT-related pathways that are connected with melatonin synthesis and circadian physiology (Tian et al., 2022). In animal models, sleep deprivation-related microbial changes have been associated with altered expression of core clock genes, including Bmal1 and Nr1d1, and with disruption of colonic motility rhythms (Wang Z. et al., 2025). Mendelian randomization studies have also reported associations between genetically predicted abundance of selected microbial taxa and sleep-related outcomes. For example, genetically predicted Lachnospiraceae abundance was associated with higher risk estimates for selected sleep disorders, whereas Coprococcus-related traits were associated with more favorable sleep outcomes in the analyzed datasets (Yan et al., 2024). These genetic findings provide causal clues but do not establish that the same effects occur uniformly across populations or clinical sleep disorders.

#### 4.2.3 Relationship between microbiota metabolites and sleep regulation

Microbial metabolites, including SCFAs, bile acids, and tryptophan derivatives, are important signaling components of microbiota–gut–brain communication. Microbial conversion of tryptophan can influence indole signaling, kynurenine metabolism, enterochromaffin 5-HT production, and downstream GABAergic and melatonin-related pathways (Feng et al., 2023). Human observational studies have reported altered serum metabolites in individuals with sleep disorders, while meta-analyses of probiotic and paraprobiotic trials suggest modest improvements in selected sleep-quality outcomes in some populations (Hua et al., 2020; Yu et al., 2024). These findings do not demonstrate that improvement is uniformly mediated by restoration of a single metabolite.

The proposed relationship between intestinal gas production and sleep requires cautious interpretation. The cited clinical study evaluated breath hydrogen and methane in obstructive sleep apnea but did not demonstrate that methane directly acts on a brainstem sleep-regulatory center or prolongs rapid eye movement sleep (Kim et al., 2022). Therefore, the available evidence does not support a direct effect of intestinal methane on brainstem sleep regulation or rapid eye movement sleep. Overall, microbial metabolism provides plausible targets for sleep-related research, but the relevant metabolites, effective interventions, and responsive patient populations require further definition.

### 4.3 Role of the gut microbiota in the pathogenesis of constipation

#### 4.3.1 Characteristics of gut microbiota in patients with constipation

Multiple 16S rRNA sequencing and metagenomic studies have reported differences in microbial diversity, taxonomic abundance, and metabolic potential between patients with chronic constipation and comparison groups. In women of reproductive age with functional constipation, one study reported higher Bacteroidetes, lower Proteobacteria, a reduced Firmicutes-to-Bacteroidetes ratio, and lower abundance of butyrate-producing taxa including Roseburia and Fusicatenibacter (Li et al., 2021). Metagenomic analysis of slow-transit constipation identified higher abundance of *Gordonibacter pamelaeae* and *Bifidobacterium longum* and lower abundance of *Coprococcus comes* and *Roseburia intestinalis* in the studied cohort (Han K. et al., 2024). These findings describe cohort-specific patterns and should not be interpreted as a universal microbial signature because functional constipation, slow-transit constipation, IBS-C, medication-related constipation, and Parkinson’s disease-associated constipation have distinct clinical backgrounds and potential mechanisms.

A bidirectional Mendelian randomization study reported that genetically predicted Coprococcus abundance was associated with a lower risk estimate for constipation, whereas genetically predicted Bacteroidetes abundance was associated with a higher risk estimate (Yu et al., 2024). These findings provide genetic evidence consistent with potential causal relationships, but they remain dependent on the validity of the genetic instruments and the taxonomic resolution of available microbiome genome-wide association studies. Together with observational evidence, they suggest that selected microbial functions may contribute to constipation through effects on intestinal motility, secretion, barrier function, and inflammatory signaling.

#### 4.3.2 Gut microbiota and gastrointestinal motility

The gut microbiota participates in the regulation of gastrointestinal motility through interactions among microbial metabolites, the enteric nervous system, smooth muscle cells, interstitial cells of Cajal, enteroendocrine signaling, and immune activity. Dysbiosis may therefore contribute to the initiation or persistence of transit-related dysfunction, but slowed intestinal transit can also alter the luminal environment and reshape microbial composition. SCFAs, bile acids, and 5-HT-related pathways are among the principal microbial and host signals implicated in peristalsis and secretion. Reduced SCFA availability may be associated with impaired colonic transit, barrier dysfunction, and inflammatory changes (Zhang et al., 2021; Zheng et al., 2025). In a loperamide-induced mouse model, a probiotic combination containing *Lacticaseibacillus paracasei* JY062 and *Lactobacillus gasseri* JM1 improved gastrointestinal motility-related outcomes, altered gastrointestinal hormones, increased interstitial cells of Cajal, and modified microbial composition and fecal SCFAs (Cheng et al., 2023). These findings support a mechanistic effect in the studied animal model but should not be generalized directly to human constipation. Conversely, loperamide-induced motility delay itself can reduce microbial richness and diversity and increase selected taxa, illustrating the bidirectional relationship between transit and microbial ecology. Parkinson’s disease medications may also influence intestinal motility and microbiota composition, as shown in experimental models (van Kessel et al., 2022).

#### 4.3.3 Interactions between microbiota metabolites and the enteric nervous system

Microbial metabolites are important messengers within the gut–brain axis and can influence enteric sensory, motor, secretory, and glial functions. SCFAs such as butyrate and propionate provide energy for colonocytes and activate free fatty acid receptors on epithelial and enteroendocrine cells, thereby influencing 5-HT release, enteric neuronal activity, and peristalsis (Ma et al., 2023; Zheng et al., 2022). Altered bile acid metabolism, including changes in secondary bile acids such as deoxycholic acid, may also affect receptor-mediated signaling and colonic motility (Zheng et al., 2025). Most peripheral 5-HT is produced by enterochromaffin cells, while the gut microbiota can modify tryptophan availability and host 5-HT-related metabolism rather than simply serving as a direct source of circulating serotonin.

**Figure 2:**
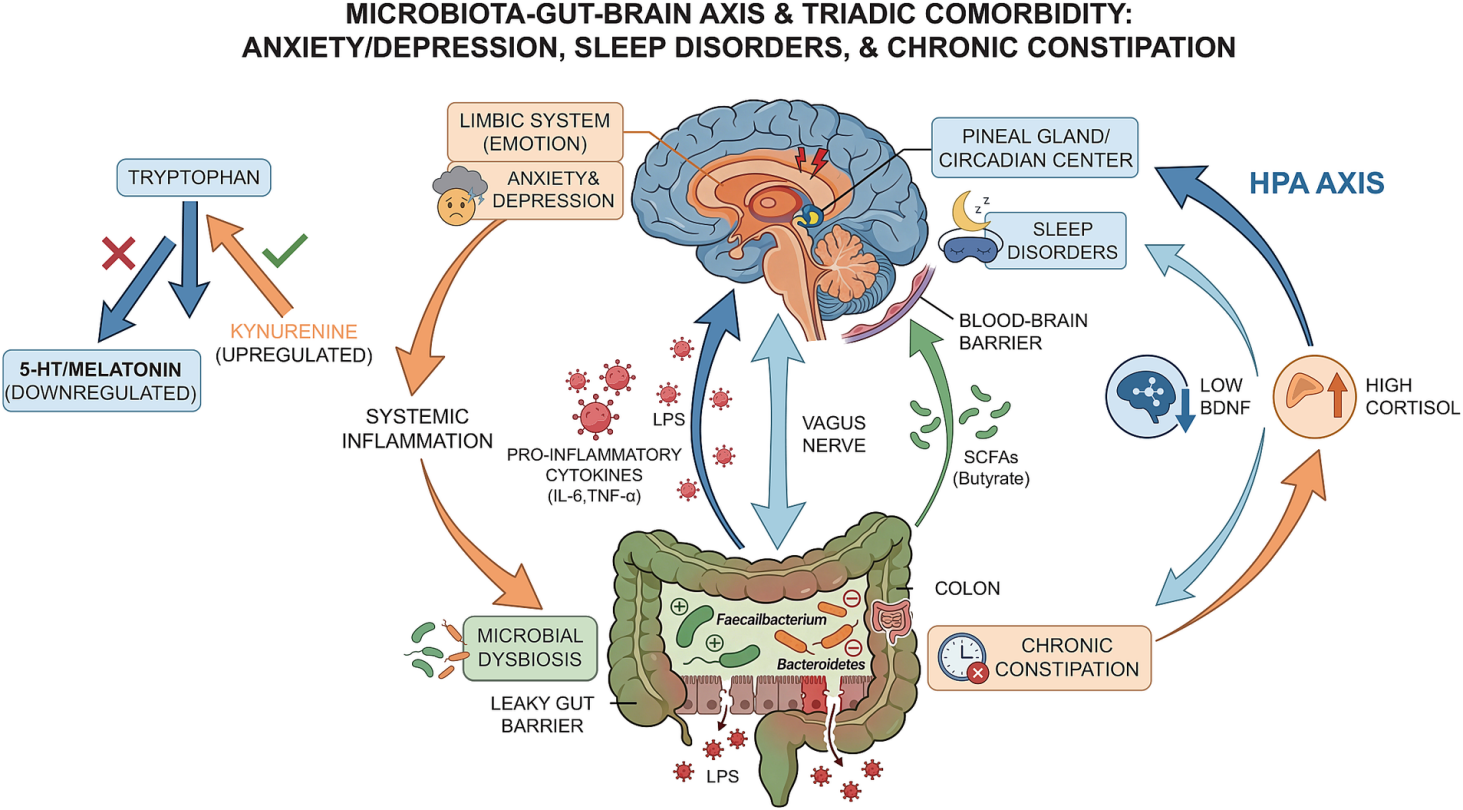
The microbiota–gut–brain axis as an integrative framework for the triadic comorbidity of anxiety/depression, sleep disorders, and chronic constipation. The figure summarizes reciprocal relationships involving microbial dysbiosis, intestinal barrier dysfunction, inflammatory signaling, microbial metabolites, vagal communication, HPA axis activity, emotional regulation, sleep–wake processes, and gastrointestinal motility. HPA, hypothalamic–pituitary–adrenal; LPS, lipopolysaccharide; SCFAs, short-chain fatty acids; 5-HT, 5-hydroxytryptamine; BDNF, brain-derived neurotrophic factor. The figure was independently drawn and integrated by the authors using Adobe Illustrator (Adobe Inc., San Jose, CA, United States), following commonly used schematic representations of medical and biological organs, cells, and signaling pathways. The figure provides a conceptual synthesis of the evidence reported in the cited literature. Arrows indicate reported or proposed biological relationships and do not imply that all depicted relationships represent established direct causality in human studies.

In a preclinical study, an engineered 5-HT-producing *Escherichia coli* Nissle strain improved gastrointestinal motility and selected behavioral outcomes in experimental models (Li et al., 2022). A recent bioRxiv preprint reported that microbiota-derived succinate promoted enteric nervous system regeneration in adult mice after antibiotic exposure (Aydin et al., 2024). This preprint provides preliminary evidence restricted to the reported animal model, and the relevance of this finding to human chronic constipation requires further validation. Collectively, these studies support the capacity of microbial metabolites to modulate enteric neuronal and glial processes, while their relevance to human chronic constipation requires clinical validation (Figure 2).

## 5 Comorbidity of anxiety/depression, sleep disorders, and constipation and its clinical significance

### 5.1 Epidemiological associations

Epidemiological studies indicate substantial co-occurrence among affective symptoms, sleep disturbances, and chronic constipation across several clinical populations. In Parkinson’s disease, approximately 44.1% of patients with constipation in one cohort also exhibited rapid eye movement sleep behavior disorder (RBD), and their coexistence was associated with more rapid cognitive and motor decline (Woo et al., 2024). In children with autism spectrum disorder (ASD), constipation and sleep disturbances frequently co-occur, and preliminary observational studies of washed microbiota transplantation have reported improvement in both stool-related and sleep-related outcomes (Zhang et al., 2022). In schizophrenia, cross-sectional mediation analysis indicated that constipation severity statistically mediated the relationship between affective symptoms and sleep quality (Huang et al., 2025). These findings demonstrate clinically relevant co-occurrence but do not by themselves establish a single shared cause. They support investigation of interacting neural, behavioral, gastrointestinal, and microbiota-related processes.

### 5.2 Bidirectional clinical relationships among the three conditions

The clinical relationships among anxiety, depression, sleep disorders, and chronic constipation appear to be bidirectional and population-dependent. In functional gastrointestinal disorders, anxiety and depressive symptoms are more prevalent than in the general population (Ballou et al., 2023). Among patients with functional constipation, those with prominent somatic symptoms showed higher GAD-7 and PHQ-9 scores and more severe constipation than those without prominent somatic symptoms (Zhao et al., 2024). Constipation is also a common non-motor symptom of Parkinson’s disease and is associated with depression, anxiety, and RBD (Chen et al., 2023). In people with schizophrenia, constipation severity statistically mediated the association between affective symptoms and sleep quality, suggesting that gastrointestinal symptoms may contribute to the clinical relationship between emotional distress and impaired sleep (Huang et al., 2025). Because these studies are mainly cross-sectional or disease-specific, they do not establish a universal sequence from one symptom domain to another. Nevertheless, they indicate that emotional symptoms, sleep disturbance, and constipation can reinforce one another and should be evaluated jointly in patients with overlapping complaints.

### 5.3 Impact on quality of life

The coexistence of anxiety, depression, sleep disorders, and constipation is associated with a substantial reduction in quality of life. Among patients with lung cancer receiving platinum-based chemotherapy, constipation was associated with greater fatigue, anxiety, depression, sleep disturbance, and lower EORTC QLQ-C30 scores (Chen H. et al., 2021). In Japanese patients with chronic constipation, poor sleep quality was accompanied by more severe constipation, higher anxiety and depression scores, and lower quality-of-life scores than good sleep quality (Yamamoto et al., 2021). In older adults, statistical mediation analyses have further suggested that stress, anxiety, and depression may partly account for the relationship between constipation and sleep quality (Papi et al., 2023). These observations support comprehensive assessment and management of overlapping symptom domains rather than isolated treatment of a single complaint.

### 5.4 Gut microbiota as a shared biological interface

Accumulating evidence suggests that gut microbial composition and function may constitute an important biological interface linking inflammatory, metabolic, neuroendocrine, and gastrointestinal processes across anxiety/depression, sleep disorders, and chronic constipation. Human studies of depression have identified microbial and metabolic profiles associated with inflammatory and lipid-related biomarkers, while sleep and constipation studies have reported overlapping alterations in SCFA production, barrier-related pathways, and microbial metabolites (De Kluiver et al., 2021). These patterns support functional convergence, although they do not establish a single microbiota configuration shared by all three conditions.

In children with ASD, washed microbiota transplantation was associated with improvements in constipation and sleep-related outcomes, together with increased α-diversity and changes in selected taxa such as Bifidobacterium (Pan et al., 2025). This observation indicates that microbiota modulation may influence multiple symptom domains in a defined clinical population, but it does not directly confirm a universal mechanism or treatment effect. SCFAs, tryptophan metabolites, bile acids, inflammatory mediators, and neuroactive precursors may connect intestinal physiology with emotional and sleep-related processes. Accordingly, disruption of microbial homeostasis may contribute to the reciprocal interaction among affective symptoms, sleep disturbance, and constipation, while remaining one component of a broader neurobiological and clinical network (Lin et al., 2024).

### 5.5 The gut–brain axis as an integrative hub in the triadic relationship

The gut–brain axis is a bidirectional network connecting cognitive and emotional brain regions with intestinal motility, secretion, immunity, barrier function, and microbial activity. Neural pathways involving the vagus nerve and enteric nervous system, endocrine pathways involving the HPA axis, and immune and metabolic signaling together provide an integrative framework for the reciprocal relationships among anxiety/depression, sleep disorders, and constipation. Chronic stress and affective symptoms may alter autonomic and neuroendocrine activity, thereby influencing visceral sensitivity, intestinal motility, mucosal barrier integrity, and microbial ecology (Chen Q. Y. et al., 2021). Conversely, constipation-associated physiological changes and microbial dysbiosis may generate neural, immune, and metabolic signals that influence emotional and sleep-related processes.

Mendelian randomization and mediation analyses have reported genetic evidence consistent with a potential effect of depression on chronic constipation, with sleep disorders proposed as a partial mediator in the analyzed datasets (Wang J.-X. et al., 2025). These findings provide causal clues but do not demonstrate a fixed clinical sequence in every patient. The coexistence of constipation, RBD, and affective symptoms in Parkinson’s disease similarly illustrates disease-specific dysfunction across central and gastrointestinal systems (Zhang X. et al., 2025). Brain-directed interventions such as repetitive transcranial magnetic stimulation have shown improvement in selected gastrointestinal and affective outcomes in functional bowel disorders, although the evidence remains condition- and protocol-specific (Li et al., 2023). Overall, the gut–brain axis serves as a central integrative framework rather than a single established causal pathway for this triadic comorbidity.

## 6 Regulatory mechanisms of the microbiota–gut–brain axis

### 6.1 Neural and autonomic pathways

The vagus nerve is a major neural route connecting the gastrointestinal tract with the central nervous system, but microbiota–brain communication also involves enteric, spinal, autonomic, endocrine, and immune pathways. Preclinical studies indicate that selected Lactobacillus and Bifidobacterium strains can influence GABA-related signaling, enteroendocrine activity, and vagal afferent responses. In the *Lactobacillus rhamnosus* JB-1 model, vagotomy abolished selected behavioral and central GABA receptor-related effects, supporting vagal dependence within that animal model (Lu et al., 2024). These findings do not imply that all probiotic effects in humans are mediated exclusively through the vagus nerve.

In Parkinson’s disease research, vagal pathways have been discussed in relation to gastrointestinal α-synuclein biology and non-motor symptoms, including constipation and RBD. However, clinical associations between constipation and RBD do not directly demonstrate retrograde α-synuclein transport as the responsible mechanism (Chen et al., 2023). In ASD, microbiota-related changes have been proposed to interact with amygdala function and sleep or emotional symptoms, but this evidence remains primarily mechanistic and preclinical (Fang et al., 2025). Together, these observations identify neural and autonomic signaling as an important component of the microbiota–gut–brain axis while highlighting the need to distinguish direct experimental evidence from clinical association.

### 6.2 Immune and inflammatory mechanisms

Gut microbial imbalance and intestinal barrier dysfunction may contribute to systemic low-grade inflammation and thereby influence neurobiological homeostasis. Microbial products such as LPS can activate pattern-recognition receptors, including TLR4, and promote cytokine signaling, endothelial activation, and microglial responses in experimental models. These pathways have been associated with anxiety- and depression-like behavior in animals, but they should not be interpreted as direct evidence that peripheral LPS uniformly causes human anxiety or depression.

Clinical conditions characterized by impaired intestinal and hepatic barrier function provide additional evidence of this interaction. In cirrhosis, endotoxemia and systemic inflammation may aggravate depressive and anxiety symptoms (Zimbrean and Jakab, 2025). In an animal FMT study, microbiota from chronic unpredictable mild stress-exposed mice activated complement C3/CR3-related signaling and microglia-mediated synaptic pruning in recipient germ-free mice, resulting in depression-like behavioral changes (Hao et al., 2024). Human metabolomic studies have also identified inflammatory profiles, including increased glycoprotein acetylation, in depression, although these profiles differ from those observed in anxiety disorders (De Kluiver et al., 2021). Collectively, immune and inflammatory pathways are important mediators within the microbiota–gut–brain axis, but their contribution varies across diseases and evidence types.

### 6.3 Microbial metabolic and neuroendocrine pathways

Microbial metabolites, including SCFAs, bile acids, and tryptophan derivatives, can influence intestinal, immune, neuroendocrine, and neural processes. SCFAs interact with enteric neurons, epithelial cells, immune cells, and enteroendocrine pathways and may facilitate colonic transit and defecatory reflexes (Dalile et al., 2019; Wang et al., 2023; Zhou et al., 2024). Human studies of depression have reported reduced abundance or functional capacity of selected SCFA-producing taxa. Experimental administration of butyrate has been associated with restoration of hippocampal BDNF-related signaling and improvement of depression-like behavior in animal models; however, these preclinical findings do not establish therapeutic efficacy in patients.

The gut microbiota also contributes to bile acid deconjugation and conversion to secondary bile acids. These metabolites can activate the farnesoid X receptor (FXR) and Takeda G protein-coupled receptor 5 (TGR5), thereby influencing intestinal secretion, motility, immune activity, and enteric glial function (Zheng et al., 2025). In chronic constipation, altered bile acid metabolism may contribute to disrupted colonic transport rather than serving as a single sufficient cause (Zhang et al., 2021). HPA axis dysregulation and altered tryptophan–kynurenine metabolism have also been associated with depression, anxiety, insomnia, and microbial disturbance (Han Z. et al., 2024; Schatzberg et al., 2014; Lieberman et al., 2016; Marx et al., 2021). Together, these pathways provide biologically plausible links between psychological stress and gastrointestinal dysfunction and support the development of mechanism-informed interventions.

## 7 Advances in microbiota-targeted interventions

### 7.1 Microbiota-targeted therapies

#### 7.1.1 Application of probiotics and prebiotics

Probiotics and prebiotics have shown potential for modulating gut microbial composition and function and for improving selected emotional, sleep-related, and gastrointestinal outcomes. The vagus nerve represents one of the neural pathways through which microbiota-related signals may communicate with the central nervous system. In mice, administration of Lactobacillus JB-1 enhanced mesenteric nerve activity, providing preclinical support for the involvement of neural signaling in microbiota–gut–brain communication (Lu et al., 2024). Selected Lactobacillus and Bifidobacterium strains have also been investigated for their potential effects on anxiety and depressive symptoms through changes in SCFA production, intestinal barrier function, and HPA axis activity; however, these effects appear to be strain-, population-, and study-design-dependent (Zhang J. et al., 2025). At the strain level, *Bifidobacterium longum* CECT 30763 alleviated depressive- and anxiety-like behaviors in a chronic social defeat mouse model (Tamayo et al., 2025), whereas *Bifidobacterium longum* 1714 reduced anxiety-like behavior in another preclinical model, with microglial regulation proposed as a possible contributing mechanism (Savignac et al., 2014). Prebiotics, including selected oligosaccharides and inulin, may promote the growth or metabolic activity of beneficial bacteria and thereby support intestinal barrier and gastrointestinal functions. In patients with functional constipation, prebiotic interventions were associated with increased fecal abundance of Bifidobacterium and improvements in defecation frequency and stool characteristics (Lévano et al., 2025; Rawi et al., 2020). The combined administration of probiotics and prebiotics as synbiotics may provide complementary effects; however, additional well-designed clinical trials are required to establish their efficacy across different diseases and symptom combinations (Martins et al., 2024).

#### 7.1.2 Potential benefits of fecal microbiota transplantation

FMT has been investigated as a microbiota-modifying intervention in selected refractory gastrointestinal and neurological conditions. Clinical studies in patients with constipation-predominant irritable bowel syndrome have reported improvements in colonic transit-related outcomes following FMT. These effects have been associated with changes in microbial diversity and the abundance of butyrate-producing bacteria, although the specific mechanisms and durability of these changes remain to be clarified (Li et al., 2024). In children with ASD, FMT was associated with improvements in constipation symptoms and reductions in Sleep Disturbance Scale for Children scores (Zhang et al., 2022). In animal models, FMT from healthy donors has also been reported to increase hippocampal BDNF expression and alleviate depression-like behaviors, providing preclinical support for a potential microbiota-related contribution to behavioral regulation (Ding et al., 2023). Nevertheless, the clinical use of FMT for anxiety or depressive disorders remains exploratory. Standardized donor screening, preparation procedures, administration protocols, long-term follow-up, and safety evaluation are therefore required before broader clinical application.

### 7.2 Lifestyle modifications

Dietary patterns, physical activity, and sleep timing may influence emotional, sleep-related, and gastrointestinal outcomes through both microbiota-dependent and microbiota-independent pathways. Time-restricted eating has been associated with increased bowel movement frequency and improved sleep-related outcomes in patients with constipation, with restoration of microbial and host circadian rhythms proposed as a potential contributing mechanism (Li J. et al., 2025). Among Japanese elementary school students, insufficient sleep was associated with an increased risk of constipation, whereas regular physical activity was associated with a lower risk, supporting a relationship among sleep behavior, physical activity, and gastrointestinal function (Furuya et al., 2025). Dietary patterns can also influence gut microbial composition and metabolic activity. Mediterranean-style dietary patterns, characterized by a high intake of dietary fiber and polyunsaturated fatty acids, have been discussed in relation to sleep and metabolic health; however, the extent to which these effects are specifically mediated by SCFA-producing bacteria or reductions in systemic inflammation requires further clarification (St-Onge and Zuraikat, 2019). Among young Japanese women, higher daily dietary fiber intake was associated with more favorable constipation, depressive symptom, and sleep-related outcomes (Honda et al., 2025). Although dietary fiber may support gastrointestinal motility and microbial fermentation, individual tolerance, baseline diet, and disease subtype should be considered. In patients with IBS, low-FODMAP dietary interventions may reduce fermentable substrate availability and alleviate symptoms such as bloating and difficult defecation, although microbiota responses differ among individuals (Ghega et al., 2025). Polyphenolic compounds may additionally influence microbial production of neuroactive metabolites, including tryptophan derivatives, and may therefore provide potential targets for future precision-nutrition strategies (Li H. et al., 2025). Collectively, lifestyle modification represents an important supportive component of integrated management, but its effects should not be attributed exclusively to changes in the gut microbiota.

### 7.3 Disease-specific combined and adjunctive interventions

Disease-specific combinations of conventional pharmacotherapy and microbiota-directed interventions have been explored as adjunctive approaches for patients with overlapping emotional, sleep-related, and gastrointestinal symptoms. In constipated patients with anxiety or depressive symptoms, the combined use of selected antidepressants and probiotics has been associated with improvements in emotional symptoms and sleep quality, although the contribution of each treatment component requires further clarification (Hongyu et al., 2025). In Parkinson’s disease, disease-specific pharmacotherapy such as rasagiline and probiotic-based approaches have been investigated in relation to non-motor symptoms, including sleep disturbance and constipation. These findings should be interpreted within the specific clinical context of Parkinson’s disease rather than generalized to patients with primary anxiety, depression, insomnia, or chronic constipation (Schrempf et al., 2018; Yin and Zhu, 2022). In sleep-deprived mice, a prebiotic formulation containing short-chain galacto-oligosaccharides and long-chain fructo-oligosaccharides at a ratio of 9:1 increased fecal SCFA concentrations and reduced inflammatory and anxiety-like responses associated with sleep deprivation (Chung et al., 2023). These findings support continued investigation of adjunctive strategies, but do not yet establish broadly applicable synergistic treatment protocols for the three clinical domains.

### 7.4 Emerging microecological and neuromodulatory approaches

**Figure 3:**
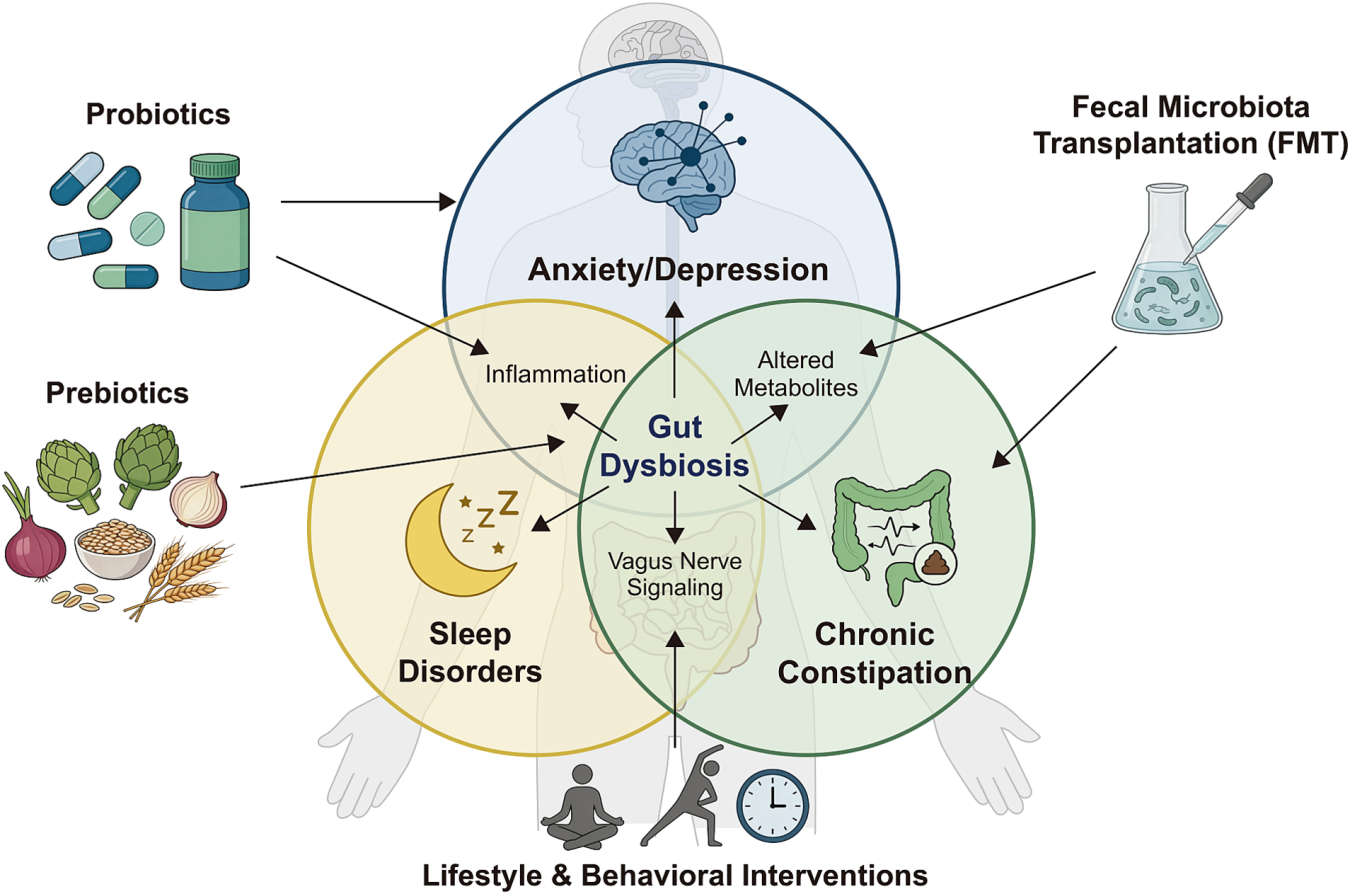
Microbiota-targeted and adjunctive interventions for the triadic comorbidity of anxiety/depression, sleep disorders, and chronic constipation. The figure summarizes probiotics, prebiotics, fecal microbiota transplantation, and lifestyle or behavioral interventions that may influence microbial dysbiosis, inflammatory signaling, microbial metabolites, vagal pathways, and overlapping symptom domains. FMT, fecal microbiota transplantation. The figure was independently drawn and integrated by the authors using Adobe Illustrator (Adobe Inc., San Jose, CA, United States), following commonly used schematic representations of medical and biological organs, cells, and signaling pathways. The figure provides a conceptual synthesis of the evidence reported in the cited literature. Arrows indicate reported or proposed biological relationships and do not imply that all depicted relationships represent established direct causality in human studies.

In addition to probiotics, prebiotics, dietary modification, and FMT, emerging microecological approaches such as postbiotics and phage-based strategies have attracted increasing attention. Postbiotics consist of inactivated microorganisms, microbial components, or microbial metabolites and may offer practical safety and stability advantages in selected settings; however, their effects are product-specific and should not be assumed to be equivalent to those of live biotherapeutics. Long-term supplementation with heat-inactivated *L. gasseri* CP2305 in healthy adults was associated with reductions in anxiety-related symptoms, improvements in sleep quality, and changes in gut microbial composition (Lin et al., 2024). Phage therapy provides a theoretically precise strategy for targeting selected bacterial populations, although its application to emotional disorders, sleep disorders, or chronic constipation remains at an early stage and requires careful evaluation of ecological and safety consequences (Sahu et al., 2023). Bioactive compounds derived from traditional medicines, including berberine hydrochloride, have also been investigated for their potential to influence microbial composition, intestinal barrier function, and inflammation-related pathways. Nevertheless, their effects on the microbiota–gut–brain axis should be interpreted as multi-component and context-dependent rather than as direct inhibition of a single abnormal pathway (Wang et al., 2024). Non-invasive neuromodulatory approaches, including transcutaneous vagus nerve stimulation, may additionally influence gastrointestinal and neurobehavioral outcomes through bidirectional gut–brain communication. Evidence that these effects are specifically mediated by changes in microbiota composition remains preliminary (Báez-Suárez et al., 2023) (Figure 3; Table 1).

**Table 1:** Comparative summary of microbiota-related evidence across anxiety/depression, sleep disorders, and chronic constipation.

Condition	Representative microbiota findings	Major metabolites or pathways	Main evidence types	Representative interventions
Anxiety and depression	Reduced abundance of selected SCFA-producing taxa and enrichment of inflammation-associated taxa have been reported in some populations, although taxonomic findings remain heterogeneous	SCFAs, tryptophan–kynurenine metabolism, intestinal barrier function, immune-inflammatory signaling, HPA axis, and vagal pathways	Human observational studies, animal models, Mendelian randomization analyses, and preliminary clinical interventions	Selected probiotics, prebiotics, dietary modification, FMT, and adjunctive interventions
Sleep disorders	Changes in microbial diversity and in the abundance of selected SCFA-producing or inflammation-associated taxa have been reported, but findings differ according to sleep phenotype and study method	SCFAs, tryptophan-derived metabolites, 5-HT and melatonin-related pathways, circadian clock regulation, inflammation, and HPA axis activity	Human observational studies, sleep-deprivation animal models, Mendelian randomization analyses, and limited interventional studies	Probiotics, postbiotics, dietary and circadian interventions, physical activity, and neuromodulatory approaches
Chronic constipation	Alterations in microbial diversity and selected taxa, including butyrate-producing bacteria, have been reported in functional and slow-transit constipation, although no universal taxonomic signature has been established	SCFAs, bile acids, 5-HT-related signaling, enteroendocrine pathways, enteric nervous system activity, and gastrointestinal motility	Human observational studies, animal models, Mendelian randomization analyses, and clinical intervention studies	Prebiotics, probiotics, synbiotics, dietary fiber, low-FODMAP approaches in selected patients, and FMT

Reported microbial alterations vary across studies because of differences in diagnostic criteria, diet, medication exposure, geographic background, sequencing platforms, sample types, and bioinformatic pipelines. Taxonomic findings should therefore not be interpreted as universally reproducible disease-specific microbial signatures. Evidence derived from animal models, observational human studies, Mendelian randomization analyses, and clinical interventions represents different levels of mechanistic and causal inference. SCFAs, short-chain fatty acids; HPA axis, hypothalamic–pituitary–adrenal axis; 5-HT, 5-hydroxytryptamine; FMT, fecal microbiota transplantation.

## 8 Future research directions

### 8.1 From association to mechanism

Future research should further clarify the molecular processes through which gut microbial functions interact with neural, immune, endocrine, and metabolic systems. Much of the current evidence remains associative, making it difficult to determine the temporal sequence between microbial alterations and host responses. Longitudinal studies with repeated sampling and functional measurements are therefore required to distinguish whether microbial changes act as causes, consequences, or components of reciprocal regulation. Specifically, the proposed relationship among constipation-associated hydrogen sulfide (H2S), amygdala neuronal activity, and epigenetic regulation, including histone deacetylation-related processes, requires direct experimental validation (Hou et al., 2025). Furthermore, integrated multi-omics approaches combining metagenomics, metabolomics, host transcriptomics, immune profiling, and neuroimaging may help identify biologically connected pathways within the microbiota–immune–neuroendocrine network (Zeng et al., 2025). Such studies should also incorporate standardized sample collection, control of dietary and medication-related confounding, correction for multiple testing, and independent validation in external populations.

### 8.2 Precision interventions and predictive modeling

Precision phenotyping based on microbiome characteristics represents an important direction for the development of individualized interventions. Among children with ASD, those with constipation symptoms showed lower baseline microbial diversity than their non-constipated counterparts. Variations in early responses to WMT further suggest that the effects of microbiota-directed interventions may differ according to individual clinical and microbial characteristics (Pan et al., 2025). Future studies should therefore consider baseline microbial composition and function, dietary background, medication exposure, disease subtype, comorbidity pattern, bacterial strain, intervention dose, and treatment duration.

Machine-learning models may assist in predicting responses to particular probiotic strains, dietary plans, or other microbiota-directed interventions by integrating indicators such as SCFA-producing capacity and bile acid metabolic profiles. However, model development should include strict separation of training and validation datasets, control of batch effects, prevention of overfitting, external multicenter validation, and assessment of clinical interpretability (Fei et al., 2025). Sex- and gender-related differences should also be considered when designing and interpreting microbiota-based and sleep-related interventions (Angell et al., 2025). Rather than assuming uniformly greater sensitivity among women, future studies should prespecify sex- and gender-stratified analyses and examine whether these factors influence baseline symptoms, treatment access, adherence, and response patterns (Angell et al., 2025).

### 8.3 Interdisciplinary clinical translation

Clinical translation will require coordinated collaboration among psychiatry, sleep medicine, gastroenterology, microbiology, nutrition, and data science. An integrated “microbiome–clinical–digital health” platform may support longitudinal assessment of microbial features, sleep quality, emotional symptoms, physical activity, and gastrointestinal function. Wearable devices may provide repeated measurements of sleep and activity patterns, while fecal microbiota and metabolite analyses may provide complementary biological information. However, these measurements should be integrated through prospectively defined clinical protocols rather than assumed to constitute an automatically effective closed-loop intervention system (Zeng et al., 2025).

Although it is not specific to microbiome research, the multicenter cooperative model represented by the International Progressive Multiple Sclerosis Alliance provides a general organizational example for data harmonization, shared research infrastructure, and translation from mechanistic findings to clinical application (Thompson et al., 2022) Future studies should establish standardized clinical outcomes, microbial sampling and processing methods, intervention quality-control procedures, and long-term safety monitoring. Patient-reported outcomes (PROs) may be incorporated alongside objective psychological, sleep-related, gastrointestinal, and microbiological measurements to support individualized treatment adjustment and longitudinal assessment (Li J. et al., 2025).

## 9 Limitations and translational considerations

The current evidence base includes animal models, human observational studies, Mendelian randomization analyses, and preliminary clinical interventions. These approaches provide complementary information but address different research questions and should not be regarded as equivalent forms of mechanistic or causal evidence. Animal models are particularly valuable for investigating biological pathways; however, germ-free animals exhibit developmental alterations in immune, metabolic, and nervous system function, whereas antibiotic-treated models may be affected by host responses that are not specific to microbial depletion. In addition, anxiety-like and depression-like behaviors measured in rodents do not fully reproduce the clinical, cognitive, and social complexity of human psychiatric disorders.

The translation of FMT findings also requires caution. Differences in donor selection, sample preparation, route of administration, dosage, recipient characteristics, and microbial engraftment may substantially influence treatment outcomes. FMT transfers not only bacteria but also viruses, bacteriophages, microbial metabolites, and other biological components. Therefore, an observed effect cannot automatically be attributed to a particular bacterial taxon or metabolite. Long-term safety, stability of engraftment, potential transmission of undesirable biological traits, and disease-specific risk–benefit profiles require further evaluation.

Human observational studies are affected by reverse causality and multiple sources of confounding. Diet, psychotropic medication, laxative use, antibiotics, probiotics, age, sex, geographical location, body mass index, disease severity, and comorbid conditions may influence both microbial composition and clinical symptoms. Stool samples may not fully represent mucosa-associated microbial communities, while differences in sequencing platforms, amplified regions, reference databases, and bioinformatic pipelines may contribute to inconsistent taxonomic findings. These limitations make it difficult to define a universally reproducible microbial signature for anxiety, depression, sleep disorders, or chronic constipation.

Interventional studies also remain heterogeneous in bacterial strain, dose, formulation, treatment duration, control condition, clinical outcome, and follow-up period. Improvements in emotional, sleep-related, or gastrointestinal symptoms do not necessarily demonstrate that the clinical effects were mediated by changes in the gut microbiota. Future trials should therefore measure microbial and metabolic changes together with prespecified clinical outcomes and should evaluate whether the observed biological changes mediate or predict symptom improvement.

## 10 Conclusion

Anxiety and depressive symptoms, sleep disorders, and chronic constipation frequently coexist and may interact in ways that increase symptom burden and reduce quality of life. Their clinical relationships involve overlapping neural, neuroendocrine, immune, metabolic, and gastrointestinal processes. Within this context, the microbiota–gut–brain axis provides a central integrative framework for understanding reciprocal interactions among emotional regulation, sleep–wake processes, intestinal physiology, microbial metabolism, and host inflammatory responses.

Current evidence supports this framework at several complementary levels. Animal studies provide mechanistic evidence that microbial manipulation can influence selected behavioral, immune, metabolic, and gastrointestinal outcomes. Human observational studies demonstrate clinical associations among microbial features, emotional symptoms, sleep disturbances, and constipation but cannot independently establish temporal or causal direction. Mendelian randomization analyses provide genetic evidence consistent with potential causal relationships, although their interpretation depends on instrument validity and the quality of the underlying microbial datasets. Clinical intervention studies provide preliminary evidence that selected probiotics, prebiotics, dietary strategies, postbiotics, and FMT may improve specific outcomes in defined populations. However, the findings remain heterogeneous and cannot yet be generalized across all patients.

The available evidence is limited by variation in study populations, diagnostic criteria, medication and dietary exposure, sample collection, sequencing methods, microbial interventions, and outcome measures. Findings obtained from germ-free animals, antibiotic-treated models, animal FMT experiments, and behavioral tests should not be directly generalized to human psychiatric disorders. Similarly, clinical improvements observed after microbiota-directed interventions do not by themselves establish that microbial changes mediated the treatment effects. Future research should combine longitudinal clinical assessment with standardized microbiome, metabolomic, immune, and neurobiological measurements and should include independent validation and long-term safety evaluation.

From a clinical perspective, microbiota-targeted approaches should not replace established psychiatric, sleep-medicine, or gastrointestinal treatments. Nevertheless, they may provide useful adjunctive strategies and research directions for the integrated management of patients with overlapping emotional, sleep-related, and gastrointestinal symptoms. Clinicians should therefore consider possible comorbid symptoms rather than evaluating anxiety, depression, sleep disturbance, or constipation in complete isolation. A multidisciplinary approach involving psychiatry, sleep medicine, gastroenterology, microbiology, nutrition, and data science may help identify clinically meaningful patient subgroups and support the development of safer and more individualized interventions.

## Glossary

5-HT: 5-hydroxytryptamine (serotonin)
GABA: γ-aminobutyric acid
SCFAs: Short-chain fatty acids
ENS: Enteric nervous system
FMT: Fecal microbiota transplantation
BDNF: Brain-derived neurotrophic factor
NTS: Nucleus tractus solitarius
GLP-1: Glucagon-like peptide-1
PYY: Peptide YY
IL-6: Interleukin-6
TNF-α: Tumor necrosis factor-α
AhR: Aryl hydrocarbon receptor
HDAC: Histone deacetylase
IDO1: Indoleamine 2,3-dioxygenase 1
HPA axis: Hypothalamic–pituitary–adrenal axis
LPS: Lipopolysaccharide
BBB: Blood–brain barrier
STC: Slow-transit constipation
ICCs: Interstitial cells of Cajal
RBD: Rapid eye movement sleep behavior disorder
ASD: Autism spectrum disorder
FC: Functional constipation
PD: Parkinson’s disease
SOM+: Somatic symptom-positive group
rTMS: Repetitive transcranial magnetic stimulation
H2S: Hydrogen sulfide
WMT: Washed microbiota transplantation
PROs: Patient-reported outcomes
